# Analysis of Genetic Variation across the Encapsidated Genome of *Microplitis demolitor* Bracovirus in Parasitoid Wasps

**DOI:** 10.1371/journal.pone.0158846

**Published:** 2016-07-08

**Authors:** Gaelen R. Burke

**Affiliations:** Department of Entomology, University of Georgia, Athens, Georgia, United States of America; Oklahoma State University, UNITED STATES

## Abstract

Insect parasitoids must complete part of their life cycle within or on another insect, ultimately resulting in the death of the host insect. One group of parasitoid wasps, the ‘microgastroid complex’ (Hymenoptera: Braconidae), engage in an association with beneficial symbiotic viruses that are essential for successful parasitism of hosts. These viruses, known as Bracoviruses, persist in an integrated form in the wasp genome, and activate to replicate in wasp ovaries during development to ultimately be delivered into host insects during parasitism. The lethal nature of host-parasitoid interactions, combined with the involvement of viruses in mediating these interactions, has led to the hypothesis that Bracoviruses are engaged in an arms race with hosts, resulting in recurrent adaptation in viral (and host) genes. Deep sequencing was employed to characterize sequence variation across the encapsidated Bracovirus genome within laboratory and field populations of the parasitoid wasp species *Microplitis demolitor*. Contrary to expectations, there was a paucity of evidence for positive directional selection among virulence genes, which generally exhibited signatures of purifying selection. These data suggest that the dynamics of host-parasite interactions may not result in recurrent rounds of adaptation, and that adaptation may be more variable in time than previously expected.

## Introduction

**Fig 1:**
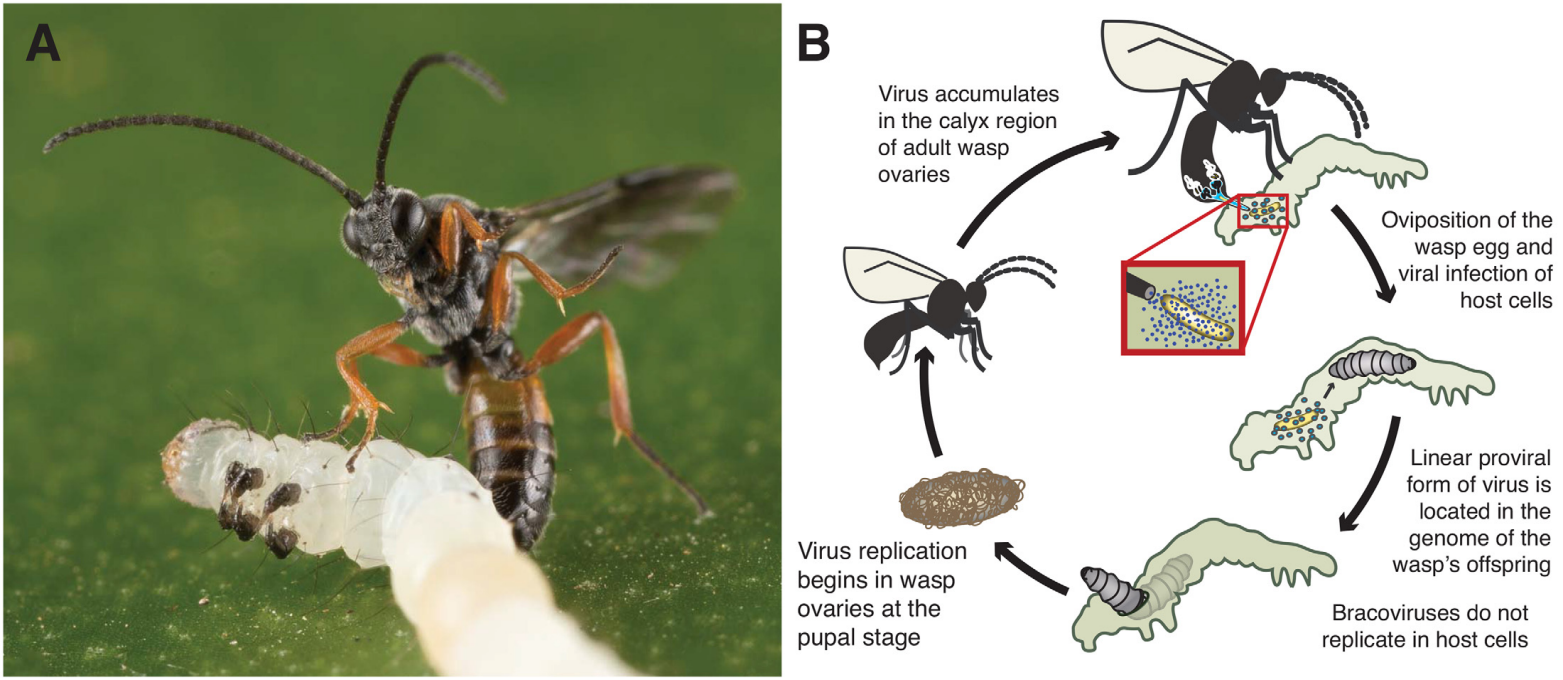
The life cycle of the wasp *M*. *demolitor* and its bracovirus. (A) A female wasp parasitizing a caterpillar host (*Chrysodeixis includens*), (B) a life cycle demonstrating wasp oviposition of eggs and virus into caterpillar hosts, transmission of the proviral form of the virus, and timing and tissue tropism of viral replication in wasps but not in host cells.

What traits are meaningful for the success or failure of parasites? For insects that feed upon other insects, such as parasitoids, several factors are important, ranging from the ability to find and detect hosts to the effectiveness of factors that counteract host defenses. Hymenopteran endoparasitoids develop internally in hosts and must resist host defense mechanisms, making factors that promote survival inside hosts key players in interactions between parasitoids and hosts. Endoparasitoids have a ‘parasitism arsenal’ comprised of one or more products such as venom, teratocytes, ovarian proteins and beneficial viruses that are injected into hosts to promote parasitism [1,2]. Viruses can be of major importance for successful parasitism in wasp lineages, and have been acquired multiple times during the evolution of several species-rich groups of parasitoid wasps [3–6]. One group of wasps, the ‘microgastroid complex’, is a monophyletic assemblage of an estimated 50,000 species belonging to six subfamilies within the family Braconidae that all employ viruses named Bracoviruses (BVs) during parasitism of lepidopteran hosts (moths and butterflies) (Fig 1) [7,8]. BVs persist in an integrated form in the wasp’s genome, and are activated to replicate in the calyx region of ovaries in the pupal stage of development [9–11]. As adults, female wasps oviposit eggs, venom and BV particles into caterpillar hosts. BVs then enter host cells and rapidly transcribe virulence genes that alter host immunity and physiology to promote growth and survival of developing wasp progeny [12,13]. The BV genome is comprised of multiple, unique proviral segments that form circular dsDNAs that are singly encapsidated (packaged into virions) to form a mixed, non-equimolar pool of virions [9,14–17]. BVs do not replicate in hosts because the genes required for replication have been transferred to regions of the wasp genome that are not excised or packaged into virions [10,18]. The virus and wasp form a mutually beneficial relationship because actions of BV virulence genes are required for successful completion of the parasitic stage of wasps’ development, and the survival of wasp progeny is the only route for transmission of the integrated virus.

BVs evolved in the ancestor of the microgastroid complex through acquisition of a virus similar to nudiviruses and baculoviruses [10]. The architecture and gene content of BV encapsidated genomes is distinctly different from their viral ancestors. Sequence data from four wasp species (*Cotesia congregata*, *Glyptapanteles flavicoxis*, *G*. *indiensis*, and *Microplitis demolitor*) and their associated BVs named CcBV, GfBV, GiBV and MdBV show that proviral segments are organized into several loci [18–20]. Some of these loci contain tandemly arranged segments whereas others contain a single segment. BV genomes have G + C content and gene density more similar to insect than to viral genomes [21]. Many BV virulence genes belong to gene families, several of which have been biochemically characterized and are known to target multiple major pathways in the hosts’ immune system [13]. Among BV genomes, gene content is highly dynamic and duplication, recombination, gain and loss of whole segments, genes or gene families are common [11,22]. In contrast, the genomic locations of viral replication genes (including those encoding protein components of virions such as nucleocapsid and envelope proteins) have only been characterized for *M*. *demolitor*, and are dispersed among wasp genes across a large portion of the wasp genome, separate from the proviral segments [18].

Three features of BVs and BV-carrying wasps have led to the hypothesis that virulence genes are engaged in ‘arms race’ dynamics with host immune genes to maintain successful parasitism. First, BV virulence gene products serve as an interface between parasitoid and host in their direct interaction with the host immune system, and second, the dynamic nature of BV genes and segments among species is suggestive of rapid or adaptive evolution. Finally, groups of BV-carrying wasps are very species-rich and host-specific, which could be due to adaptations to promote or avoid parasitism among species [23–26]. The current dogma on molecular evolution in host-pathogen associations suggests that signatures of arms race dynamics should be present in BV genes in the form of recurrent selective sweeps and amino acid replacements in protein-coding genes [27,28]. Under this hypothesis, the genes of key importance in host/parasite interactions would be among the fastest-evolving genes, which could be identified (if unknown) using tests for positive directional selection. A limited number of studies from two wasp genera have used BV genome sequencing to identify signatures of positive selection in virulence genes with mixed results [20,29,30]. In this study, data was generated for a third genus of BV-carrying wasps to look for adaptive evolution acting upon genes in the wasp species *Microplitis demolitor*, including polymorphism data from laboratory and field-collected wasps, and the BV genome from the sister species to *M*. *demolitor*, *Microplitis mediator* (Fig 1). The results revealed an overall absence of positive directional selection acting upon BV virulence genes, suggesting re-evaluation of the hypothesis about the evolution of genes involved in interactions between offensive viruses and parasitoid hosts is warranted.

## Results

### Viral genome re-sequencing reveals substantial variation in field compared to laboratory populations

In this study, the genetic variability in the MdBV genome from *Microplitis demolitor* wasps was characterized. Viral genome variation was described from four different samples; two from laboratory populations maintained in culture for approximately 30 years, and two from field populations. The two laboratory population samples differed in the time of sampling and the method of sample DNA preparation. Both laboratory population samples were generated during the recently completed *M*. *demolitor* genome sequencing project [18,31], so were already available for the analysis of variation within the MdBV genome. The first sample (hereafter “pooled laboratory 1”) was isolated and amplified from viral DNA extracted from a pool of wasp ovaries in August of 2011, whereas the second sample (“pooled laboratory 2”) was sequenced directly from genomic DNA isolated in May of 2013 from a second pool of ovaries when viral replication was at its peak, taking advantage of the strong overrepresentation of viral genomic DNAs in wasp ovaries. Samples from field populations were also prepared to enrich for viral DNA, with one sample representing a pool of 12 wasp individuals (hereafter “pooled field”) and the other representing a single individual (hereafter “individual field”).

The portion of the *M*. *demolitor* genome sequence containing viral genome segments is 277,539 bp in aggregate size and is spread across eight loci. The MdBV genome includes 25 proviral segments (named A through K and K1 to X) [18]. Locus 1 (13 segments), 2 (five segments) and 3 (two segments) contain multiple segments while loci 4–8 each contain one. Each locus containing segments is amplified as a unit, followed by excision of individual segments (except for the two segments in Locus 3, N and J, which are amplified separately, [31]). Each viral genome segment contains Wasp Integration Motifs located at both ends of the segment that mark the sequence for circularization and demarcate segments from non-viral regions of the wasp genome. Individual segments range in size from 5,575 to 17,325bp, with mean size 11,413bp. The MdBV proviral genome used in this study contained a total of 95 protein-coding virulence genes. These wasp population samples described above and the reference genome provided the prerequisites for analysis of variation within the MdBV genome. It is important to note that this reference-based approach may not identify large insertions or deletions such as gene duplication or loss.

To characterize viral sequence variation, the MdBV genome was re-sequenced from the four samples above using Illumina technology (S2 Table). The number of quality-filtered reads sequenced for each sample ranged from eight to 75 million (S2 Table). Between 21 and 85% of reads could be successfully mapped to the viral genome reference sequence (S2 Table). As each segment is present at non-equimolar abundance in ovaries and virus particles, and as Illumina sequence reads are sequenced in proportion to the presence of their originating DNA in a sample, sequence coverage varied by viral genome segment. Average coverage per site in sequence pileups for viral segments ranged from 85x for Segment W in the individual field sample to 7864x for Segment J in the first pooled laboratory sample (S3 Table).

All DNA samples were taken after viral genome amplification had already occurred in wasp ovaries, so polymorphisms sequenced could represent i) real variation within the viral portion of the wasp genome, ii) differential amplification of allelic variants of viral genome segments, or iii) errors that are produced during the amplification process. Allele frequencies in the individual field population were analyzed to identify any biases caused by differential amplification of alleles present in diploid female chromosomes during virus replication. If alleles are amplified equally, then heterozygote alleles should have mean allele frequencies of 50%. For all genomic loci containing MdBV proviral segments that are co-amplified, the alternative allele frequencies for heterozygotes had means centered around 0.5, and while the mean frequencies were significantly different among loci for 1077 single nucleotide polymorphisms (SNPs), Tukey-Kramer’s Honestly Significant Difference (HSD) comparison did not reveal distinct differences between loci (S1 Fig).

The total number of segregating sites in field population samples was an order of magnitude higher than laboratory population samples (Table 1). In pooled and individual field samples, 5435 and 1707 sites (1.96–0.62% of sites, respectively) were variable compared to the reference. In contrast, variation was detected for only 132 and 142 sites (0.05% of sites) in pooled laboratory samples. Of these sites, approximately half (50/132 and 29/142) appeared to be fixed differences between the laboratory samples and the reference sequence. Given that both of the laboratory samples and the reference genome were generated from the same laboratory population, these sites either represent errors in the reference sequence or sites that are truly polymorphic in the population with variation not sampled in the pooled wasp individuals used for DNA preparation and sequencing. A smaller proportion of sites were fixed with respect to the reference in field populations (119/5435 and 630/1707). The pooled field population had 49 multi-allelic sites (sites with more than one alternative allele compared to the reference), while the individual field sample contained nine and the pooled laboratory samples had one and zero multi-alleleic sites for populations 1 and 2, respectively. Only five or six indels were observed in the laboratory populations compared to 44–51 in the viral genomes of wasps from the field population. Although all efforts were made to pool DNA from individual wasps equally, it is possible that the pooling approach resulted in uneven sampling of alleles among individuals, which may have lead to slight underestimation of the total amount of variation present in the pooled population samples. However, it is likely that the majority of variant sites were identified given the high depth of coverage achieved during sequencing. After analysis of the total amount of variation within different population samples, I next sought to examine where variants were present with respect to virulence genes.

**Table 1:** Number of SNPs by type of segregating site. The total number of segregating sites is comprised of the number of polymorphic, fixed, multi-allelic and insertion or deletion sites.

Population	Total number of segregating sites	SNPs	Indels	Number of polymorphic sites	Fixed sites different from reference
Pooled field	5435	5391	44	5316	119
Individual field	1707	1656	51	1077	630
Pooled laboratory 1	132	127	5	82	50
Pooled laboratory 2	142	136	6	113	29

### Most genetic variation within wasp populations is silent with respect to gene function

As MdBV genes are extremely important for wasps’ parasitism success, any variation in viral coding sequences could potentially influence the function of viruses in lepidopteran hosts. However, the MdBV genome and BV genomes in general are relatively gene sparse, with coding density ranging from 17–33% compared to 67–94% in the related nudiviruses and baculoviruses [11]. BV genes often do not have canonical viral or eukaryotic gene structure; many genes are small and contain short introns. Untranslated regions (UTRs) (as annotated) are based upon read mapping from transcript-sequencing datasets, and given that they were not defined by more direct measures of transcription initiation or termination, annotated UTRs were ignored in this study and regions 300bp upstream and downstream of coding regions of genes were used to serve as proxies for UTRs instead. SNPs were assigned to functional categories based upon the types of changes they represent in the MdBV proviral genome. Counts for each category were allowed to sum to more than the number of segregating sites because several situations are considered separately: 1) alleles within multiallelic sites, 2) sites that fall into shared regions of overlapping genes on different strands, and 3) sites that are located in the upstream or downstream regions of multiple closely-spaced genes.

For both laboratory and field populations, the majority of segregating sites (78–80%) were found in intergenic regions (Table 2). The remaining segregating sites fell within exons (8–13%) or introns (8–14%). These proportions were significantly different from the overall percentage of sites belonging to these categories in the MdBV genome (73% intergenic, 19% exonic, 8% intronic) for all populations except the second pooled laboratory population. All indels occurred in intergenic or intronic regions, so no frameshift variants were observed. Overall, most sequence variation within MdBV proviral segments was located in regions that have no known function, and few variant sites were identified within the coding sequences of virulence genes. Despite the low number of variants present in protein-coding regions of the MdBV genome (compared to neutral expectations), my hypothesis predicts that arms race evolutionary dynamics act upon these regions and will result in selective sweeps and amino acid replacement changes in virulence genes. Variants within MdBV virulence genes were further analyzed using both within- and between-species comparisons to look for evidence of positive directional selection.

**Table 2:** The number of SNPs in the MdBV proviral genome by annotation class and genomic location.

Substitution category	Substitution type	Percentage of MdBV genome	Pooled field ***	Individual field ***	Pooled laboratory 1 *	Pooled laboratory 2 N.S.
Intergenic		73%	4338 (79%)	1373 (80%)	106 (78%)	119 (80%)
	Downstream		575	188	6	7
	Upstream		511	155	6	7
Exons		19%	715 (13%)	217 (13%)	11 (8%)	16 (11%)
	Non-synonymous coding		398	117	4	9
	Synonymous coding		301	97	7	7
	Synonymous stop		1	1	0	0
	Start lost		3	0	0	0
	Stop gained		9	1	0	0
	Stop lost		3	1	0	0
Introns		8%	460 (8%)	137 (8%)	19 (14%)	13 (9%)
Splice site acceptor or donor		<1%	7	0	0	0

*p < 0.05 *X*^*2*^ *test*,

***p < 0.0001 *X*^*2*^ *test* for rejecting hypothesis that the number of SNPs is proportional to the cumulative length (in bp) of each available genomic substitution site type in the MdBV genome.

### Most MdBV genes are experiencing purifying selection

**Fig 2:**
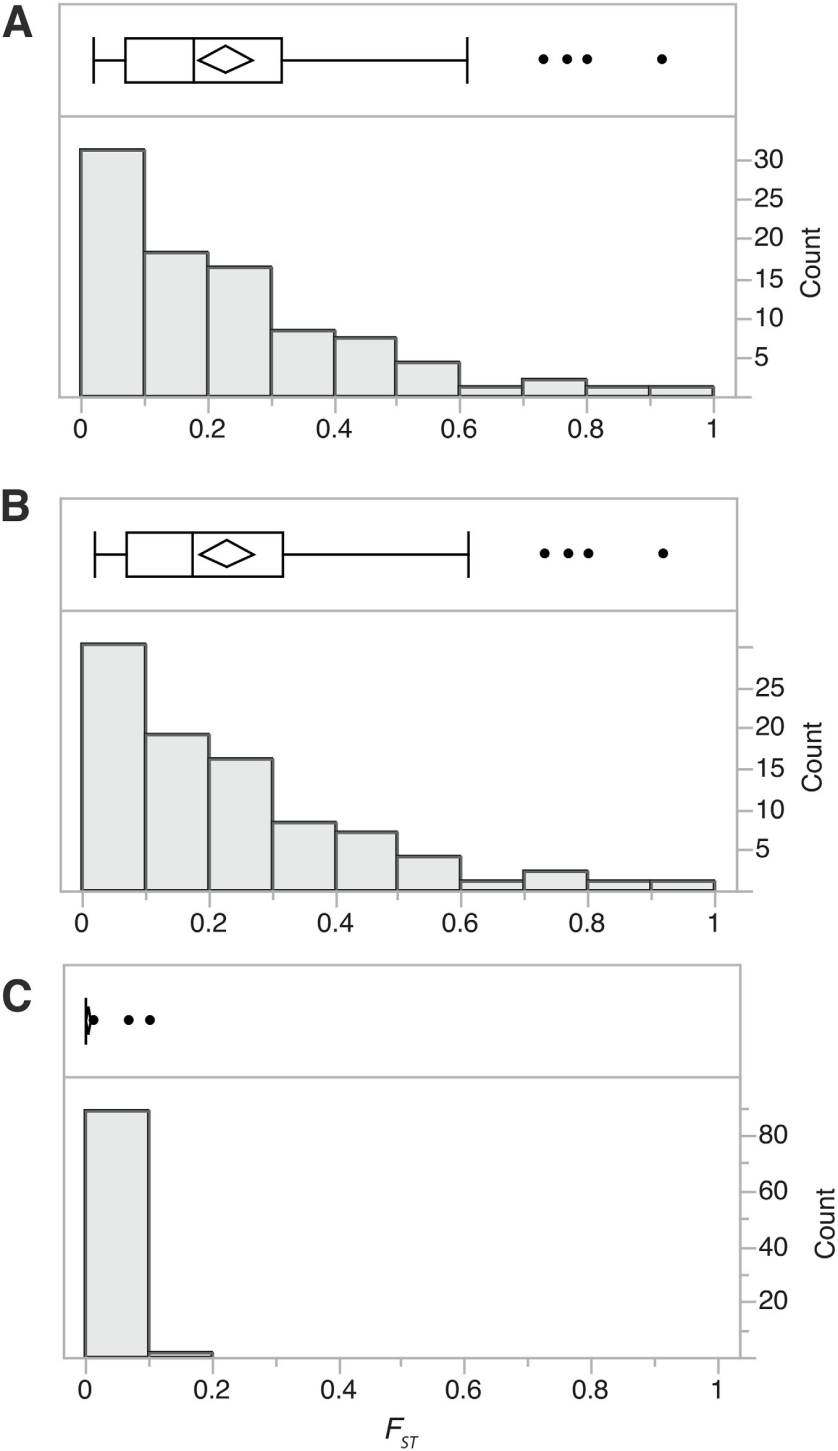
Sub-population differentiation (*F*_*ST*_ values) for MdBV proviral genes (N = 61) for the following comparisons: A) Pooled field vs. laboratory population 1, B) Pooled field vs. laboratory population 2, C) Laboratory population 1 vs. laboratory population 2. The upper portion of each panel contains a box plot showing the mean and standard error (diamond shape) and quartiles (25–75% shown as a box) of *F*_*ST*_ values across genes, while outliers are depicted as single points. The lower portion contains a histogram of *F*_*ST*_ values across genes, with counts shown on the axis to the right.

Three measures were employed to detect selection acting upon BV virulence genes: *F*_*ST*_, *dN/dS*, and the McDonald-Kreitman test. *F*_*ST*_ between the two laboratory and the pooled field subpopulations was measured for each gene as a unit to identify any genes with significant differentiation in SNPs between subpopulations. 89/95 genes had SNPs within exons with a mean of 7.9 SNPs per gene. *F*_*ST*_ values for all genes were examined to identify any outlier genes that may be more differentiated between subpopulations compared to other genes due to selection for genetic diversification or relaxation of selection. The distributions of *F*_*ST*_ values across genes for the pooled field population compared to either laboratory population 1 or 2 revealed four outliers: *ank-N4*, *ptp-V2*, *ptp-H3* and *orph-X5* (Fig 2A, 2B and S4 Table). For all four genes, SNPs were identified in the pooled field population only. In contrast, most *F*_*ST*_ values for genes between the two laboratory subpopulations were equal to zero except for three outliers, *glc1*.*8*, *orph-G2* and *orph-K1*. All genes except *ank*-*N4* contained at least one non-synonymous polymorphism. These genes represent candidates for further studies to determine whether these changes are a product of differential directional selection between these sub-populations.

Two more powerful tests for selection, *dN/dS* and the McDonald-Kreitman test, were employed to detect positive directional selection among MdBV genes by examining patterns of variation within protein-coding regions of virulence genes in MdBV and the BV from a sister species *Microplitis mediator*, MmBV. *M*. *mediator* may be more of a generalist species compared to *M*. *demolitor*, with broad palearctic distribution and the ability to parasitize approximately 40 different species of genera from the Noctuidae family of the Lepidoptera [32]. Both *M*. *demolitor* and *M*. *mediator* can parasitize the same laboratory noctuid moth hosts with similar success rates. The non-equimolar abundance and repetitive DNA present in BV genomes has made full genome sequencing difficult, requiring a combination of wasp genome sequencing (to control for coverage, and to characterize integrated proviral genome architecture) and deep sequencing of viral encapsidated DNAs to identify proviral regions of the wasp genome [18–20]. To generate comparative data between BV-carrying wasp species without sequencing the full *M*. *mediator* wasp genome, a partial genome sequence for *Microplitis mediator* Bracovirus (MmBV) was generated using deep sequencing of viral encapsidated DNAs only. In the MmBV genome assembly, full-length matches were identified for 14 MdBV segments (two fully assembled and 12 in two or more fragments), while partial matches were identified for ten segments, and a match to one MdBV segment could not be identified. A total of 56 MmBV genome fragments (contigs) contained 94 genes that were potential orthologs of MdBV genes.

**Fig 3:**
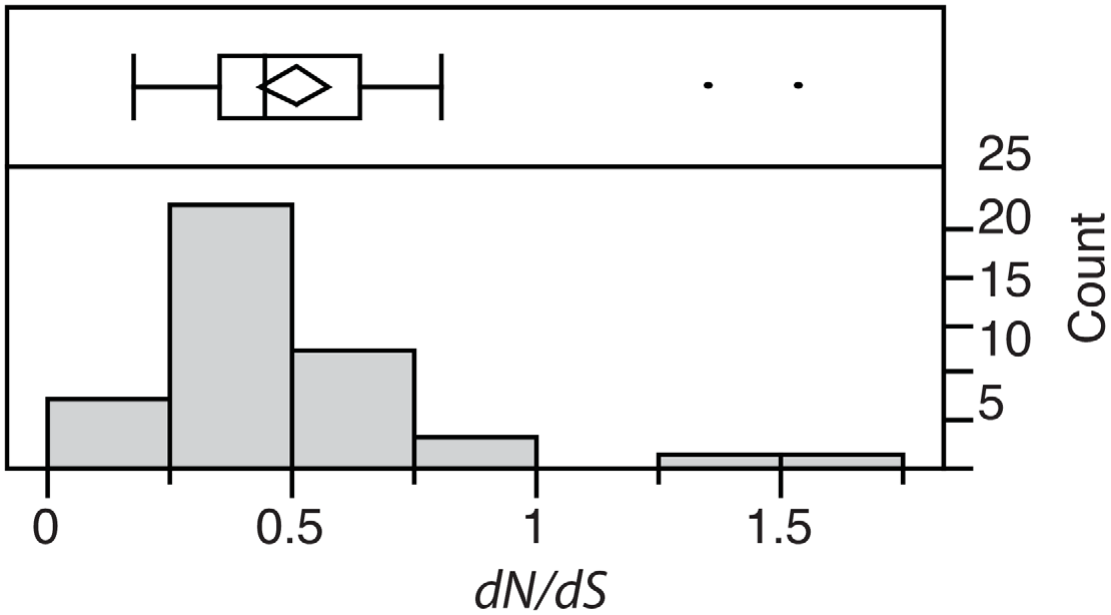
Distribution of *dN/dS* values per gene between MdBV and MmBV orthologs (N = 51). The upper and lower panels are the same as for Fig 2.

Orthologs between MdBV and MmBV were identified using BLAST and reciprocal best hits, or phylogenetic analysis of the *ptp* and *ank* gene families (S2 Fig). In several cases orthologs could not be identified for *ptp* or *ank* family members due to duplications in either MdBV or MmBV. Unfortunately, the fragmented nature of the MmBV genome made it impossible to use genome synteny to infer orthology for gene family members for which orthology could not be resolved by phylogenetic analysis. Given the focus upon *M*. *demolitor* in this work and the incomplete assembly of the MmBV genome, full comparison of gene content between these sister species will be reserved for another study. It was possible to identify 51 orthologs between MdBV and MmBV, which had average *dS* values of 0.088 to 0.538, indicating that these viruses have not diverged to a point at which saturation of sites is an issue. The majority of genewise *dN/dS* ratios were less than 1 (Fig 3), which suggests that purifying selection is an important selective force between species as well as within *M*. *demolitor* populations. 34 genes had *dN/dS* < 0.5, indicative of purifying selection, while 17 genes had intermediate *dN/dS* values evolving neutrally or with relaxed selection. While two genes were outliers in the distribution of *dN/dS* values (*dN/dS* values were 1.4 and 1.5 for *orph-A1* and *orph-F2*, respectively), a *dN/dS* greater than 1.8 has been used in previous studies of BV evolution to confidently conclude a gene has been experiencing positive selection (Fig 3, [29]).

The number of polymorphic sites per protein-coding MdBV gene was too low to use the McDonald-Kreitman (M-K) test for analysis of most individual genes (S5 Table, [33]). Only one of the genes identified as *F*_*ST*_ outliers, *ptp-H3*, had enough SNPs to make an M-K test appropriate; but no significant enrichment of non-synonymous polymorphisms in the pooled field population was identified (*X*^*2*^ = 2.05, *p* = 0.152). However, the M-K test can be applied to the entire coding portion of these two *Microplitis* BV genomes (Table 3). The result of this test shows no difference in the number of non-synonymous substitutions between species compared to non-synonymous polymorphisms within species, indicating evolution not significantly different from neutral expectations across MdBV genes as a whole (Fisher’s exact test, *p* = 0.8). Following analysis of the selective forces acting upon protein-coding genes, we next took a broader approach via analysis of the entire MdBV genome to explore the selective forces acting upon non-protein coding regions.

**Table 3:** McDonald-Kreitman test for selection across coding regions within BV genes in the pooled field population. The number of synonymous (S) and non-synonymous (N) substitutions within MdBV (polymorphic) and between 51 MdBV and MmBV genes (fixed), *p* = 0.8, Fisher’s exact test.

Differences	S	N	N/S
Polymorphic	163	211	1.29
Fixed	1,670	2,099	1.26

### Levels of sequence variation differ among MdBV segments

The hypothesis predicting arms race dynamics among MdBV and host genes did not include any predictions for what to expect when examining selection upon the entire MdBV genome, which is mostly comprised of intergenic DNA. However, observed differences in the abundance, gene content, and functional importance of MdBV proviral segments have shown that some proviral segments are more important than others for contributing to parasitism success in hosts and thus may experience different forms or strength of selection as individual units. Several metrics can be used to examine patterns of variation within a genome to provide information about the kinds of selective pressures experienced by individual regions in the genome. Variation within each viral genome segment was analyzed using three metrics: 1) the raw numbers of sequence variants, 2) Tajima’s *D*, a measure of allele frequency variation, and 3) *F*_*ST*_ between sub-populations of *M*. *demolitor*. These measures can provide information about the selective forces acting upon viral genome segments.

**Fig 4:**
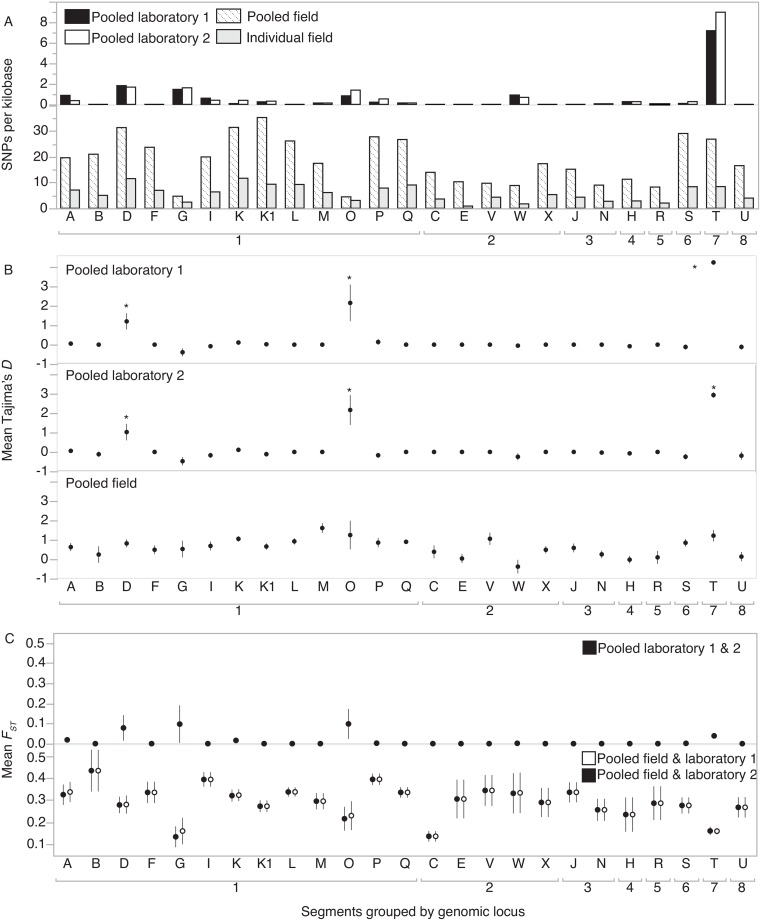
Population genetic statistics for MdBV proviral segments in the *M*. *demolitor* wasp genome. A) Variable sites per kilobase in viral genome segments in laboratory and field population samples. Samples from the laboratory subpopulation are shown in the upper panel while field population data are depicted in the lower panel. B) Estimates of Tajima’s *D* statistic across MdBV viral genome segments in three pooled subpopulation samples, averaging across non-overlapping windows of size 1000bp along segments. Least Squares Analysis of Variance (ANOVA), Pooled laboratory 1: *F*_*24*,*244*_ = 24.8, *p* < 0.0001; Pooled laboratory 2: *F*_*24*,*240*_ = 14.6, *p* < 0.0001; Pooled field population: *F*_*24*,*236*_ = 3.6, *p* < 0.0001. Asterisks indicate segments that are significantly different from all other segments with the Tukey-Kramer HSD test with the significance level alpha set to 0.01. C) Mean *F*_*ST*_ calculated across MdBV genome segments for pairwise comparisons between laboratory subpopulations (upper panel), or between laboratory and field subpopulations (lower panel), averaged across non-overlapping windows of size 1000bp along segments. Least Squares ANOVA, Pooled laboratory 1 & 2: *F*_*24*,*276*_ = 4.7, *p* < 0.0001; Pooled field & laboratory 1: *F*_*24*,*476*_ = 3.1, *p* < 0.0001; Pooled field and laboratory 2: *F*_*24*,*476*_ = 3.3, *p* < 0.0001. MdBV proviral segments are grouped into genomic loci for visualization only, and were not included as a factor in statistical analyses. Error bars represent standard errors of the mean; error bars are typically too small to be visible.

In field populations, the raw number of segregating sites in samples was variable within one order of magnitude across segments (Fig 4A). In contrast, in both laboratory samples, the number of segregating sites was skewed to fall primarily within Segment T followed by Segments D and G. Tajima’s *D* is a statistic that compares the difference between estimates of nucleotide diversity to the number of segregating sites, which in a population at neutral equilibrium should be zero. Overall, mean Tajima’s *D* for the viral genome was 0.16 for laboratory sample 1, 0.09 for laboratory sample 2, and 0.65 for the pooled field sample (Table 4). These averages were not significantly different from zero according to the confidence intervals specified in Tajima 1989 [34] (Table 4). However, analysis of mean Tajima’s *D* for individual segments revealed that segments D, O and T had statistically higher values of *D* compared to other segments in both laboratory population samples, and additionally, the Tajima’s *D* statistics for segments O and T were significantly elevated from neutral expectations (zero) (Fig 4B, Table 3).

**Table 4:** Population genetic parameters estimated from segregating sites within the MdBV proviral genome (aggregate size 277,539bp) from three population samples.

Population sample	Number of pooled individuals	Mean Tajima’s *π*	Mean Watterson’s *θ*	Mean Tajima’s *D*	95% C. I. for Tajima’s *D*	95% C.I. for null hypothesis of *D* = 0
Pooled field	12	0.0044	0.0034	0.6535	0.5533–0.7537	-1.765 to 1.979
Pooled laboratory 1	15	0.0002	0.0001	0.1564	0.0588–0.2540	-1.765 to 1.979
Pooled laboratory 2	30	0.0002	0.0001	0.0850	-0.0013–0.1713	-1.584 to 1.708

Both pooled laboratory samples and the pooled field sample represent a total of three different sub-populations of *M*. *demolitor*, separated by either space or time of sampling. Comparison between both laboratory sub-populations for differentiation in allele frequencies resulted in generally low *F*_*ST*_ values for each segment, which indicated very little differentiation between these sub-populations (Fig 4C). Comparisons between the pooled field sub-population and either of the laboratory sub-populations resulted in intermediate *F*_*ST*_ values, with significant differences between segments within genomic loci and between loci, but no major outliers.

## Discussion

This study surveyed intraspecific variation within the MdBV encapsidated genome in both laboratory and field populations of *M*. *demolitor* wasps. Few variant sites in laboratory populations were expected given the predicted loss of heterozygosity under the assumptions of the neutral theory due to population inbreeding and genetic drift associated with bottlenecks that the laboratory population has been experiencing for 30 years in culture. Given these expectations, I coupled wasp laboratory culture data with samples from field populations of wasps that were more representative of viral genome heterogeneity in natural populations. As expected, compared to the field population, an order of magnitude fewer variant sites were observed in the laboratory populations. In the field population, although more variation was observed in the MdBV encapsidated genome overall, most variant sites were located outside of protein-coding regions of virulence genes.

For variant sites within MdBV virulence genes, I looked for signatures of selection that could be indicative of arms race dynamics. Analysis of *F*_*ST*_ at the level of genes identified only four genes that had significantly differentiated between the laboratory and field populations, possibly indicating differential directional selection. Following this unexpected result, I obtained the sequences for virulence genes in *M*. *mediator* BV to perform more powerful tests for selection in MdBV using intra- and interspecies sequence comparisons. Comparison of variation within and between species across all genes with the McDonald-Kreitman test also did not support positive directional selection in the form of selective sweeps within wasp populations. Finally, the majority of *dN/dS* ratios for each gene were less than 1, indicating that purifying selection may be an important selective force between species as well as within *M*. *demolitor* populations. No *dN/dS* ratios indicating an excess of amino acid replacements in genes due to positive directional selection were identified. In total, I used several tests for selection with multiple types of data and did not find convincing evidence for positive directional selection due to arms race dynamics acting upon protein-coding regions of virulence genes. Instead, the data from this study show that purifying selection might be the major selective force acting upon viral genes.

If bracoviruses are not of key importance for host range and specificity, why do other BV genomes show signatures of adaptation between species? Two previous studies have shown that several BV genes are experiencing positive directional selection, but presented mixed evidence for arms race dynamics between BVs and hosts. One study, based upon comparison of within- and between-species variation in BV genes from *Glyptapanteles* wasps, showed evidence for positive directional selection in BV genes using *dN/dS* statistics (13% of genes had *dN/dS* greater than 1.8) and using a McDonald-Kreitman test averaging across 72 orthologous BV genes. Another study examined variation among BV genes in four congeneric species of *Cotesia* wasps, and identified no genes evolving with positive selection between species, but between 15–30% of genes showed evidence for positive selection for intraspecific comparisons of ecologically differentiated subspecies of *Cotesia sesamiae* [29]. In fact, between-species comparisons using *dN/dS* indicated that most BV genes were evolving under purifying selection in *Cotesia* species. The authors suggested that contrary to the recurrent selective sweeps predicted for genes evolving in an ‘arms race’, *Cotesia* bracovirus speciation occurs first with a period of strong positive selection followed by strong purifying selection for most genes. Although the differences in findings between these three interspecies comparisons of BV genome evolution cannot definitively be explained, it is possible that as proposed by Jancek *et al*., the timing of adaptation events could be non-constant with strong positive directional selection only occurring in the incipient stages of speciation or during a host shift and may be obscured over time.

The lack of evidence for recurrent selective sweeps occurring in MdBV protein-coding genes and mixed evidence from other species suggests that the hypothesis that protein sequence evolution is of key importance for adaptation of virulence genes to host immune proteins may not represent the entire story for MdBV. In contrast to pattern recognition receptors involved in detecting pathogens and parasitism in insects, host proteins involved in translating these signals into an immune response through signaling pathways are of major importance and are highly conserved. I speculate that some components of the insect innate immune system are so well conserved that arms race dynamics need not occur for continuance of successful parasitism. For example, the Egf protein family in MdBV prevents activation of the melanization pathway by inhibiting cleavage of prophenoloxidase by serine proteases [35–37]. Egf1.0 can inhibit melanization in species such as *Bombyx mori* and *Manduca sexta*, even though these belong to Lepidopteran families that are non-permissive for *M*. *demolitor* parasitism [35–37]. A second example is the Ank protein family, which can bind to Rel proteins (Nuclear Factor-κB (NF-κB) transcription factors) and prevent their translocation to host cell nuclei to activate transcription of immune effector genes such as antimicrobial peptides [38]. Ank proteins can bind to and inhibit Rel proteins from completely different orders of insects (*Drosophila melanogaster*, Diptera) than their native lepidopteran hosts [38,39]. With very little polymorphism in protein-coding genes from the field population collection followed by inbreeding and genetic drift due to bottlenecks, the laboratory population possessed negligible variation upon which selection could act, yet no reduction in parasitism success was observed after rearing on a new host in the laboratory [40,41]. If BV immune suppression genes and host immune signaling genes are so conserved that they will allow parasitism of many different hosts, then why aren’t most wasp species generalists with wide host ranges?

Instead of the acquisition of changes in individual amino acids within viral proteins that mediate inactivation of the host’s immune system, 1) changes in viral proteins that are involved in infection of cells and receptor binding, 2) the rapid acquisition of new genes, gene duplication and loss, and 3) evolution of promoters to encourage viral gene expression in hosts could be equally or more important for parasitism success. First, all of the BV virulence genes that encode protein components of virions (particularly viral envelope proteins) are not present in the BV encapsidated genome [10,18], and have not been examined for signatures of positive selection due to arms race dynamics with hosts that relate to receptor binding and cell entry. In opposition to this idea, data from group 1 alphabaculoviruses (related to BVs) suggest that viral host range is not limited by cell entry factors, given that these viruses can infect both insect and non-insect cells *in vitro* despite being limited to infecting Lepidoptera (moths and butterflies) *in vivo* [42–45]. Second, BV virulence gene content varies dynamically between more distantly related wasp species, which suggests that some innovation is advantageous in order to successfully parasitize hosts. Rather than accumulating changes in protein coding genes, the rare acquisition of entirely new genes from sources outside of BV genomes may be important for maintaining successful parasitism via unknown mechanisms [1]. Third, a recent study has shown that the overall strength of viral gene transcription differs dramatically between permissive and non-permissive hosts of *M*. *demolitor*, pointing towards the importance of viral gene promoters in successful parasitism of specific host species [46]. Finally, aside from the arms race adaptations proposed at the level of the viral genome above, another important level at which adaptations (that limit host range) might occur could involve behavioral or chemically-based adaptations in host finding [2]. It is possible that pre-ovipositon interactions are more important for determining host range than those that occur after parasitism.

One final and unexpected result from the analysis of variation within the *M*. *demolitor* BV genome is evidence for the distinct evolution of two of the MdBV proviral segments in the laboratory populations. Segment T, and to a lesser extent, segments D, G and O, had elevated numbers of variable sites, while segments D, O and T had higher average Tajima’s *D* values compared to other segments. Segments O and T also had Tajima’s *D* values that were significantly elevated compared to neutral expectations. While it is tempting to conclude that these metrics are evidence for positive directional selection, the sign of the Tajima’s *D* statistic (positive, negative or near zero) was very sensitive to the low frequency allele cutoff value used (data not shown). Despite this, the relative difference between most segments and O and T were robust, suggesting that evolution of these two segments is driven by different factors compared to other segments. Segment O is arguably one of the most important segments in MdBV, because it contains critical immune suppression (anti-aggregation and anti-melanization) virulence genes, which are the most highly expressed by several orders of magnitude in host hemocytes [35–37,39,47,48]. However, the fragmented assembly and repetitive nature of Segment O makes it difficult to evaluate whether the evidence for positive selection is real or artifactual. Although Segment T is the smallest segment in the MdBV genome with the lowest abundance in the pool of virions, and it does not contain any genes, PCR assays have shown that it is present in its mature circular form within the pool of MdBV virions injected into hosts upon parasitism [18]. It is possible that both segments O and T are experiencing positive directional selection upon one or more non-coding sites within the segment.

## Experimental Procedures

### Laboratory and field collections of BVs for assessment of viral genome variation

Two populations of wasps were surveyed for genetic variation in the MdBV genome. The first population was sampled in 2012 from the Darling Downs region in Queensland, Australia, where *M*. *demolitor* is endemic. *M*. *demolitor* naturally parasitizes *Helicoverpa punctigera* (also native in Australia) and *Helicoverpa armigera* (introduced to Australia from the old world and now endemic). Wasps were originally collected from this site in Australia in 1981, were introduced into the United States in 1983 and 1990, and from that time were maintained in continuous culture on the hosts *Helicoverpa virescens* and later *C*. *includens* (formerly *Pseudoplusia includens*). Over approximately 30 years, the laboratory population of *M*. *demolitor* was maintained with relatively small population sizes with regular bottlenecks, constant environmental conditions and with no introduction of new genetic variation from outbreeding. Approximately 50 females were used for parasitism of hosts each generation, which could be used as an estimate of effective population size (*N*_*e*_), and as each generation takes an average of 11 days to develop from an egg to an adult, the culture of *M*. *demolitor* has been in the laboratory for approximately 1000 generations. *M*. *demolitor* were grown in the laboratory on *C*. *includens* hosts as previously described at 27°C with a 16-h-light:8-h-dark photoperiod [49].

The second population of *M*. *demolitor* wasps were collected from the same region in Queensland, Australia in January of 2013. A total of 205 *Helicoverpa* larvae were collected from three fields (S1 Table) in Pampas, Queensland from sorghum inflorescences (panicles). Larvae were reared on a chickpea based artificial diet [50] and from these, 21 wasps emerged and were reared to adulthood, while 13 survived until DNA extraction was possible.

The *M*. *mediator* culture used for comparative genomic analyses originated from the Netherlands from pupae collected on *Mamestra brassicae* hosts. *M*. *mediator* were received at the University of Georgia in 2008, and were henceforth grown in laboratory culture as described above for *M*. *demolitor*.

### Sample preparation and sequencing

Ovaries from field collected wasps were dissected in 150 μl of 1 x DNAse buffer to remove the virus bolus from the lumen of the calyx region of ovaries, and centrifuged gently to remove any cellular debris. The pooled field DNA was isolated from 12 pooled wasp ovaries in one DNA extraction, while the individual field sample was isolated from a single wasp ovary. 2 μl of DNAse (NEB) was added and incubated at 37°C for 30 minutes to digest all free wasp and viral DNAs. After the addition of EDTA to 10mM to inactivate the DNAse, DNA was extracted by viral nucleocapsid lysis using 250 μg of proteinase K (Roche) and 2% sarkosyl followed by incubation at 62°C for 1 h and by phenol:chloroform extraction. DNA was precipitated with 0.3M sodium acetate pH 5.2, 25 μg of glycogen and isopropanol, and pellets were resuspended in 100 μl of water.

Sequence data for the first pooled laboratory sample of MdBV were available from a previous study [18] in which viral DNA was isolated from 15 individual wasps as described above. The second pooled laboratory sample was generated from whole ovaries of 30 females of mixed pupal stages 3 and 4, when viral replication is at its peak [11,31]. DNA was extracted as above for field samples but without a DNAse step to remove free wasp and episomal viral DNAs. Sequence libraries for both field and laboratory samples were prepared using the library construction and sequencing parameters listed in S6 Table.

In summary, the first pooled laboratory sample and both field samples were prepared to only sequence DNAs packaged into virions for delivery into host insects, while the second pooled laboratory sample contained a mixture of packaged viral DNAs, unpackaged viral DNAs, and a small amount of host DNA.

### Generation of reference genomes

The *M*. *demolitor* genome sequence was published previously [18], and was isolated from a single male wasp sample from the same laboratory population described above. Given that male wasps are haploid, no segregating sites were present in the reference genome sequence. The genome reference is represented by 5,168 contigs and scaffolds of 259 megabases aggregate size, sequenced to a average depth of 26x [18]. When sequenced, Segments D and O were not assembled perfectly. Segment D was broken across two scaffolds and one contig, while less than half of Segment O is present within the wasp genome assembly compared to an earlier version of the sequence derived from sequencing viral DNAs only (rather than the whole wasp genome). Segment O is highly repetitive and the version used in this study is likely a consensus of several repeated regions within the segment [18]. While some genes were annotated with UTR regions, this information is missing for approximately 70% of genes.

A partial genome sequence for *Microplitis mediator* Bracovirus (MmBV) was generated by sequencing purified viral DNAs. Viral DNA was prepared from 100 wasp ovaries using the same methods as the field sample DNA extractions above. Library construction and sequencing metadata are available in S6 Table. The MmBV genome was assembled using Trinity [51]. Trinity is a transcriptome assembler, but due to the non-equimolar abundance of MmBV segments and their corresponding uneven representation within the pool of MmBV genomic DNA sequenced, this assembler performed the best for assembly of segments. From the contigs assembled by Trinity, those that matched the MdBV genome were selected for annotation (N = 56). MAKER was used to infer gene models within the selected contigs followed by manual curation with Apollo [52,53].

### Analysis of viral genome variation

Sequencing reads were filtered to retain reads with a minimum phred equivalent quality score equivalent of 30 or greater for more than 90% of nucleotides in a read (hannonlab.cshl.edu). Library size (S6 Table) was small for the two field samples relative to read length, so to avoid quality score biases, reads from all samples were assembled with PEAR with a maximum phred equivalent quality score of 40 (v0.9.2, [54]). Illumina reads from pooled field and laboratory populations as well as a single field-collected individual wasp were mapped against the proviral segment-containing regions of the *M*. *demolitor* genome (assembly version 1.0, AZMT0000001) with bowtie 2 [55]. Samtools version 1.2 [56] was used to remove any PCR duplicates from the datasets. A pileup was generated for the resulting read alignment files with samtools mpileup, restricting the use of paired reads to those that were paired with the expected orientation and insert size, with a maximum read depth of 8000, minimum PHRED score of 30, and minimum mapping quality of 20. To control for errors that occur at low frequency in random locations, a minimum percentage cutoff of 2% was used to filter out very low frequency polymorphisms in the pooled samples for all subsequent analyses.

bcftools version 1.2 was used to identify variant sites from pileup information [57]. Information from the bcftools multi-allelic calling method in combination with raw variant data generated by ‘bcftools view’ were used to identify SNPs and indels to retain those with a minimum variant frequency of 2%, a minimum coverage cutoff of 30, and a minimum number of three reads supporting each variant. ‘bcftools filter’ was used to remove all variant sites with read position bias (-e ‘RPB<0.05').

Variant sites were assigned to categories by the predicted effect of each variant and its location using SnpEff with a database constructed from a gff3 file from the *M*. *demolitor* viral genome annotation and the viral genome sequences [18,58]. SnpEff version 3.5d was run with default parameters modified to treat 300bp on either side of the coding region of each gene as upstream and downstream regions of potential importance to gene function (eg. proxies for UTRs that may contain promoter or terminator regions).

*F*_*ST*_, a measure of subpopulation differentiation, was calculated using popoolation2 (a program similar to popoolation that analyzes differences between two or more populations) [59]. Generally, popoolation2 identifies SNPs from a raw dataset, but to be consistent with the more stringent SNP calling method used above, lower quality SNPs were removed from the popoolation2 preliminary files prior to calculation of *F*_*ST*_ to match those used as variant sites in SnpEff analysis. To detect *F*_*ST*_ for each gene as a unit, popoolation2 was run with quality filtered preliminary files and a gtf format file of gene annotations from Burke *et al*. 2014 [18], with window and step sizes much larger than the size of any gene (1,000,000 for each). Only exons were included for each gene to detect changes that could be of potential functional importance.

Orthologs for between-species analyses were identified among the resulting MmBV protein sequences and MdBV proteins using reciprocal best BLAST hits for single-copy genes. Orthologs within the multi-member *ank* and *ptp* gene families were identified using alignment with Muscle [60], alignment curation with Mesquite (http://mesquiteproject.org), and tree construction using phylogeny.fr [61]. Synonymous and non-synonymous substitution rates were estimated using *codeml* for sequence pairs implemented by PAML with codon frequencies estimated from average nucleotide frequencies at the three codon positions [62]. The numbers of synonymous (S) and non-synonymous (N) substitutions for each pair of sequences was calculated using PAML and these were used in a McDonald-Kreitman test [63].

Popoolation version 1.2.2 was used to estimate the population genetic parameters Tajima’s *π* and Watterson’s *θ* for each population sampled using pileup information and calculating the mean from windows of size 1000 and a step size of 1000 with a minimum allele frequency of 2% and minimum coverage cutoff of 30 [64]. For popoolation analyses, reads within pileup files were randomly subsampled without replacement to reduce coverage to a maximum of 300x to equalize coverage among MdBV genome segments.

## Supporting Information

S1 Fig Alternative allele frequencies for heterozygotes in the individual field sample. This chart depicts mean allele frequency for each genomic locus (in which MdBV proviral segments are co-amplified) with error bars showing one standard deviation. Segments J and N are located in the same genomic region but are amplified separately. Alternative allele frequencies are significantly different for distinct genomic loci (ANOVA, *F*_*8*,*1068*_ *=* 2.3, *p* = 0.018), but comparison of all pairs of loci with Tukey-Kramer’s HSD revealed no distinct differences between means.
(DOCX)

S2 Fig Phylogenetic resolution of relatedness between homologs belonging to two MdBV and MmBV gene families: A) The viral ankyrin (*ank*) gene family, and B) the Protein Tyrosine Phosphatase (*ptp*) gene family. Genes are named according to their origin (MdBV or MmBV) followed by the gene name. Bootstrap support values greater than 75 are shown at each node of the trees. Pairs marked in green boxes are orthologous and were used for inter-species comparisons. Orthologous relationships could not be inferred confidently for unmarked gene family members.
(DOCX)

S1 Table *Helicoverpa* larval collection details (DOCX)

S2 Table Sequence read and mapping statistics for DNA samples analyzed in this study. (DOCX)

S3 Table Average coverage of segments after sequence pileup. Each segment is present at non-equimolar abundance in ovaries and virus particles. As Illumina reads are sequenced in proportion to the presence of their originating DNA in a sample, sequence coverage varies by viral genome segments. Coverage is defined as the read depth for reads mapped to each position in the MdBV proviral genome. During sequence pileup, a maximum coverage of 8000 was implemented. The reference sequence for Segment D was split among three scaffolds or contigs, named D1 (on Mdem_scaffold_1462), D2 (on Mdem_contig_4124537) and D3 (on Mdem_scaffold_3298) here.
(DOCX)

S4 Table Counts of polymorphisms for genes with outlier *F*_*ST*_ values in sub-population comparisons. NP—non-synonymous polymorphisms; SP—synonymous polymorphisms.
(DOCX)

S5 Table Values used for a McDonald-Kreitman test from PAML for 51 BV genes in MdBV and MmBV. NP—non-synonymous polymorphisms; SP—synonymous polymorphisms; N—non-synonymous substitutions between species; S—synonymous substitutions between species.
(DOCX)

S6 Table Library construction and sequencing parameters used to sequence each sample of BV proviral DNA. (DOCX)
