# Supplementary material for: Analysis of Genetic Variation across the Encapsidated Genome of Microplitis demolitor Bracovirus in Parasitoid Wasps
Source: PLoS One. 2016 Jul 8;11(7):e0158846. doi: 10.1371/journal.pone.0158846 (PMC4938607; doi:10.1371/journal.pone.0158846)

Supplementary Figure 1. Alternative allele frequencies for heterozygotes in the individual field sample. This chart depicts mean allele frequency for each genomic locus (in which MdBV proviral segments are co-amplified) with error bars showing one standard deviation. Segments J and N are located in the same genomic region but are amplified separately. Alternative allele frequencies are significantly different for distinct genomic loci (ANOVA, *F_8,1068_ =* 2.3*, p* = 0.018), but comparison of all pairs of loci with Tukey-Kramer’s HSD revealed no distinct differences between means.


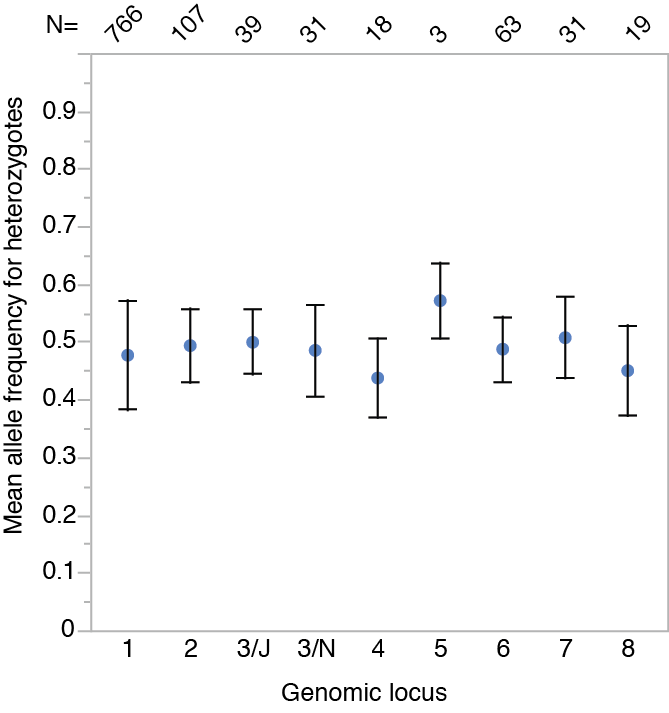

Supplement: S1 Fig — This chart depicts mean allele frequency for each genomic locus (in which MdBV proviral segments are co-amplified) with error bars showing one standard deviation. Segments J and N are located in the same genomic region but are amplified separately. Alternative allele frequencies are significantly different for distinct genomic loci (ANOVA, F8,1068 = 2.3, p = 0.018), but comparison of all pairs of loci with Tukey-Kramer’s HSD revealed no distinct differences between means. (DOCX) [file pone.0158846.s001.docx]
