# Supplementary material for: Analysis of Genetic Variation across the Encapsidated Genome of Microplitis demolitor Bracovirus in Parasitoid Wasps
Source: PLoS One. 2016 Jul 8;11(7):e0158846. doi: 10.1371/journal.pone.0158846 (PMC4938607; doi:10.1371/journal.pone.0158846)

Supplementary Figure 2. Phylogenetic resolution of relatedness between homologs belonging to two MdBV and MmBV gene families: A) The viral ankyrin (ank) gene family, and B) the Protein Tyrosine Phosphatase (ptp) gene family. Genes are named according to their origin (MdBV or MmBV) followed by the gene name. Bootstrap support values greater than 75 are shown at each node of the trees. Pairs marked in green boxes are orthologous and were used for inter-species comparisons. Orthologous relationships could not be inferred confidently for unmarked gene family members.


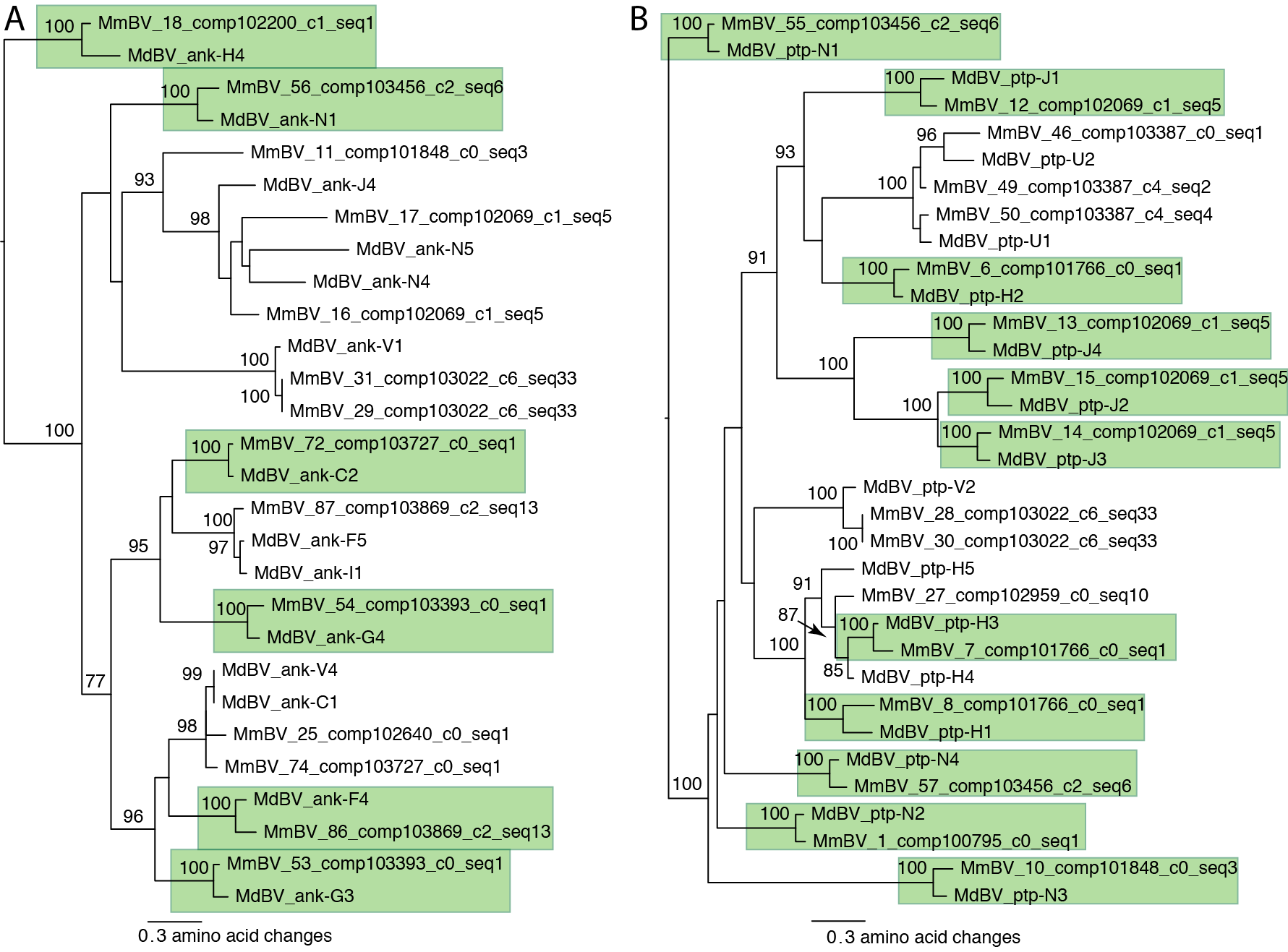

Supplement: S2 Fig — Genes are named according to their origin (MdBV or MmBV) followed by the gene name. Bootstrap support values greater than 75 are shown at each node of the trees. Pairs marked in green boxes are orthologous and were used for inter-species comparisons. Orthologous relationships could not be inferred confidently for unmarked gene family members. (DOCX) [file pone.0158846.s002.docx]
