# Supplementary material for: Analysis of Genetic Variation across the Encapsidated Genome of Microplitis demolitor Bracovirus in Parasitoid Wasps
Source: PLoS One. 2016 Jul 8;11(7):e0158846. doi: 10.1371/journal.pone.0158846 (PMC4938607; doi:10.1371/journal.pone.0158846)
Supplement: S1 Table — (DOCX) [file pone.0158846.s003.docx]

Supplementary Table 1. *Helicoverpa* larval collection details

| **Number of larvae collected** | **Location** | **Field number** | **Crop** | **Latitude** | **Longitude** |
| --- | --- | --- | --- | --- | --- |
| 75 | Pampas | P9 | Sorghum | -27.8086520 | 151.3939270 |
| 113 | Pampas | P16 | Sorghum | -27.7814557 | 151.4752582 |
| 17 | Pampas | P14 | Sorghum | -27.8138370 | 151.4475944 |
