# Supplementary material for: Analysis of Genetic Variation across the Encapsidated Genome of Microplitis demolitor Bracovirus in Parasitoid Wasps
Source: PLoS One. 2016 Jul 8;11(7):e0158846. doi: 10.1371/journal.pone.0158846 (PMC4938607; doi:10.1371/journal.pone.0158846)
Supplement: S2 Table — (DOCX) [file pone.0158846.s004.docx]

Supplementary Table 2. Sequence read and mapping statistics for DNA samples analyzed in this study.

| **Sample name** | **Number of filtered read pairs** | **Number of reads mapped to the MdBV proviral genome** | **Number of reads after duplicate removal and pair filtering** |
| --- | --- | --- | --- |
| Pooled laboratory 1 | 37,081,373 paired reads,  1,274,561 assembled reads | 31,705,715 paired reads, 877,564 assembled reads | 16,045,056 paired reads,  298,143 assembled reads |
| Pooled laboratory 2 | 14,021,890 paired reads,  443,888 assembled reads | 3,758,703 paired reads,  27,791 assembled reads | 3,095,932 paired reads,  24,358 assembled reads |
| Pooled field | 2,902,499 paired reads,  2,154,753 assembled reads | 1,770,390 paired reads,  1,478,195 assembled reads | 809,107 paired reads,  212,690 assembled reads |
| Individual field | 4,331,943 paired reads,  3,483,990 assembled reads | 266,180 paired reads,  1,719,838 assembled reads | 166,395 paired reads,  195,183 assembled reads |
