# Supplementary material for: Analysis of Genetic Variation across the Encapsidated Genome of Microplitis demolitor Bracovirus in Parasitoid Wasps
Source: PLoS One. 2016 Jul 8;11(7):e0158846. doi: 10.1371/journal.pone.0158846 (PMC4938607; doi:10.1371/journal.pone.0158846)
Supplement: S3 Table — Each segment is present at non-equimolar abundance in ovaries and virus particles. As Illumina reads are sequenced in proportion to the presence of their originating DNA in a sample, sequence coverage varies by viral genome segments. Coverage is defined as the read depth for reads mapped to each position in the MdBV proviral genome. During sequence pileup, a maximum coverage of 8000 was implemented. The reference sequence for Segment D was split among three scaffolds or contigs, named D1 (on Mdem_scaffold_1462), D2 (on Mdem_contig_4124537) and D3 (on Mdem_scaffold_3298) here. (DOCX) [file pone.0158846.s005.docx]

Supplementary table 3. Average coverage of segments after sequence pileup. Each segment is present at non-equimolar abundance in ovaries and virus particles. As Illumina sequence reads are sequenced in proportion to the presence of their originating DNA in a sample, sequence coverage varies by viral genome segments. Coverage is defined as the read depth for reads mapped to each position in the MdBV proviral genome. During sequence pileup, a maximum coverage of 8000 was implemented. The reference sequence for Segment D was split among three scaffolds or contigs, named D1 (on Mdem_scaffold_1462), D2 (on Mdem_contig_4124537) and D3 (on Mdem_scaffold_3298) here.

| **Proviral segment** | **Pooled laboratory 1 average coverage** | **Pooled laboratory 2 average coverage** | **Pooled field average coverage** | **Individual field average coverage** |
| --- | --- | --- | --- | --- |
| A | 6682 | 1919 | 673 | 173 |
| B | 7480 | 1642 | 581 | 227 |
| C | 7477 | 988 | 612 | 306 |
| D1 | 4792 | 2399 | 905 | 295 |
| D2 | 7572 | 4213 | 1052 | 293 |
| D3 | 2789 | 1358 | 709 | 220 |
| E | 7722 | 1049 | 571 | 229 |
| F | 7833 | 2346 | 1048 | 388 |
| G | 6150 | 1322 | 702 | 256 |
| H | 7728 | 4362 | 1574 | 449 |
| I | 5401 | 1487 | 624 | 189 |
| J | 7864 | 7660 | 4530 | 1429 |
| K | 3834 | 783 | 431 | 138 |
| K1 | 2855 | 583 | 358 | 106 |
| L | 7807 | 2453 | 812 | 223 |
| M | 7531 | 1784 | 896 | 229 |
| N | 7831 | 3022 | 1902 | 547 |
| O | 4668 | 3233 | 1056 | 133 |
| P | 5268 | 562 | 415 | 151 |
| Q | 5643 | 887 | 675 | 261 |
| R | 7624 | 821 | 348 | 96 |
| S | 5887 | 658 | 574 | 215 |
| T | 3954 | 304 | 166 | 108 |
| U | 7836 | 2082 | 1385 | 504 |
| V | 7596 | 1046 | 625 | 232 |
| W | 4126 | 650 | 243 | 85 |
| X | 6071 | 989 | 449 | 131 |
| All | 6223 | 1874 | 886 | 282 |
