# Supplementary material for: Analysis of Genetic Variation across the Encapsidated Genome of Microplitis demolitor Bracovirus in Parasitoid Wasps
Source: PLoS One. 2016 Jul 8;11(7):e0158846. doi: 10.1371/journal.pone.0158846 (PMC4938607; doi:10.1371/journal.pone.0158846)
Supplement: S4 Table — NP—non-synonymous polymorphisms; SP—synonymous polymorphisms. (DOCX) [file pone.0158846.s006.docx]

Supplementary Table 4. Counts of polymorphisms for genes with outlier *F_ST_* values in pooled populations. NP – non-synonymous polymorphisms; SP – synonymous polymorphisms.

| Gene name | Total SNPs in pooled populations | Types of SNPs in pooled laboratory population 1 | Types of SNPs in pooled laboratory population 2 | Types of SNPs in pooled field population |
| --- | --- | --- | --- | --- |
| *ank-N4* | 3 |  |  | 3SP |
| *ptp-V2* | 7 |  |  | 3SP, 4NP |
| *orph-X5* | 2 |  |  | 1SP, 1NP |
| *ptp-H3* | 18 |  |  | 8SP, 10NP |
| *glc1.8* | 9 | 1SP, 1NP | 1SP, 3NP |  |
| *orph-G2* | 9 | 3SP | 4SP, 1NP |  |
| *orph-K1* | 14 | 1NP | 4NP |  |
