# Supplementary material for: Analysis of Genetic Variation across the Encapsidated Genome of Microplitis demolitor Bracovirus in Parasitoid Wasps
Source: PLoS One. 2016 Jul 8;11(7):e0158846. doi: 10.1371/journal.pone.0158846 (PMC4938607; doi:10.1371/journal.pone.0158846)
Supplement: S5 Table — NP—non-synonymous polymorphisms; SP—synonymous polymorphisms; N—non-synonymous substitutions between species; S—synonymous substitutions between species. (DOCX) [file pone.0158846.s007.docx]

Supplementary Table 5. Values used for a McDonald-Kreitman test from PAML for 51 BV genes in MdBV and MmBV. NP – non-synonymous polymorphisms; SP – synonymous polymorphisms; N – non-synonymous substitutions between species; S – synonymous substitutions between species.

| **Name** | **NP** | **SP** | **N** | **S** | ***dN*** | ***dS*** | ***dN/dS*** |
| --- | --- | --- | --- | --- | --- | --- | --- |
| ank-C2 | 3 | 2 | 20 | 18 | 0.0522 | 0.1185 | 0.4404 |
| ank-F4 | 2 | 6 | 32 | 50 | 0.09 | 0.3739 | 0.2408 |
| ank-G3 | 1 | 2 | 33 | 30 | 0.0863 | 0.2101 | 0.4108 |
| ank-G4 | 0 | 1 | 33 | 26 | 0.0864 | 0.2111 | 0.4092 |
| ank-N1 | 2 | 0 | 33 | 34 | 0.103 | 0.2948 | 0.3494 |
| glc1.8 | 6 | 1 | 26 | 15 | 0.0595 | 0.1382 | 0.4305 |
| orph-A1 | 4 | 3 | 63 | 18 | 0.2446 | 0.1802 | 1.3577 |
| orph-A2 | 7 | 2 | 46 | 23 | 0.2191 | 0.2943 | 0.7444 |
| orph-A4 | 5 | 6 | 37 | 69 | 0.0717 | 0.4138 | 0.1733 |
| orph-C3 | 2 | 2 | 25 | 23 | 0.0972 | 0.1983 | 0.4904 |
| orph-C4 | 3 | 2 | 49 | 36 | 0.1368 | 0.3065 | 0.4463 |
| orph-D2 | 0 | 5 | 44 | 28 | 0.1646 | 0.2933 | 0.5612 |
| orph-D6 | 1 | 3 | 52 | 32 | 0.2415 | 0.5376 | 0.4493 |
| orph-D7 | 5 | 11 | 22 | 17 | 0.0779 | 0.1668 | 0.467 |
| orph-D8 | 13 | 4 | 49 | 24 | 0.2333 | 0.4036 | 0.578 |
| orph-E2 | 0 | 0 | 17 | 15 | 0.0667 | 0.2041 | 0.3266 |
| orph-E3 | 2 | 1 | 20 | 17 | 0.0999 | 0.2205 | 0.4532 |
| orph-F1 | 5 | 4 | 20 | 34 | 0.0735 | 0.384 | 0.1914 |
| orph-F2 | 2 | 0 | 12 | 2 | 0.1357 | 0.088 | 1.5427 |
| orph-F5 | 2 | 4 | 28 | 24 | 0.1308 | 0.3207 | 0.408 |
| orph-G2 | 3 | 6 | 104 | 57 | 0.1766 | 0.2551 | 0.6922 |
| orph-G3 | 0 | 0 | 35 | 30 | 0.113 | 0.2546 | 0.444 |
| orph-H1 | 0 | 0 | 32 | 20 | 0.1453 | 0.203 | 0.7158 |
| orph-I1 | 3 | 3 | 38 | 17 | 0.1571 | 0.1944 | 0.8079 |
| orph-K1-4 | 4 | 6 | 51 | 42 | 0.1793 | 0.4337 | 0.4134 |
| orph-K2 | 1 | 4 | 19 | 17 | 0.0935 | 0.2628 | 0.3559 |
| orph-K3 | 5 | 5 | 21 | 21 | 0.0619 | 0.1521 | 0.4068 |
| orph-L4 | 2 | 2 | 14 | 14 | 0.0952 | 0.2723 | 0.3494 |
| orph-M1 | 4 | 3 | 10 | 18 | 0.0436 | 0.1965 | 0.2221 |
| orph-M2 | 3 | 1 | 16 | 15 | 0.0705 | 0.1825 | 0.386 |
| orph-M3 | 6 | 6 | 15 | 10 | 0.0737 | 0.1703 | 0.4329 |
| orph-M4 | 0 | 2 | 20 | 8 | 0.111 | 0.1624 | 0.6835 |
| orph-M5 | 2 | 0 | 48 | 22 | 0.1991 | 0.3122 | 0.6377 |
| orph-M6 | 6 | 5 | 70 | 44 | 0.1634 | 0.3568 | 0.4578 |
| orph-M7 | 2 | 0 | 36 | 19 | 0.1424 | 0.1915 | 0.7435 |
| orph-R1 | 0 | 0 | 22 | 8 | 0.1053 | 0.1368 | 0.77 |
| orph-V3 | 1 | 2 | 26 | 15 | 0.0754 | 0.1128 | 0.6683 |
| orph-X1 | 17 | 7 | 60 | 33 | 0.1533 | 0.2237 | 0.6853 |
| orph-X2 | 17 | 2 | 55 | 54 | 0.1335 | 0.3371 | 0.3962 |
| orph-X3 | 7 | 3 | 84 | 48 | 0.2571 | 0.4052 | 0.6344 |
| ptp-H1 | 2 | 7 | 93 | 79 | 0.1778 | 0.4542 | 0.3913 |
| ptp-H2 | 3 | 6 | 58 | 89 | 0.0789 | 0.3784 | 0.2086 |
| ptp-H3 | 10 | 8 | 25 | 43 | 0.0698 | 0.3845 | 0.1815 |
| ptp-J1 | 6 | 1 | 71 | 35 | 0.1105 | 0.1373 | 0.8049 |
| ptp-J2 | 11 | 8 | 83 | 58 | 0.1281 | 0.2622 | 0.4887 |
| ptp-J3 | 13 | 2 | 57 | 44 | 0.0876 | 0.194 | 0.4513 |
| ptp-J4 | 7 | 3 | 70 | 69 | 0.1048 | 0.3042 | 0.3446 |
| ptp-N1 | 1 | 4 | 35 | 56 | 0.0516 | 0.2605 | 0.198 |
| ptp-N2 | 1 | 2 | 40 | 46 | 0.0578 | 0.1897 | 0.3046 |
| ptp-N3 | 0 | 2 | 74 | 43 | 0.1063 | 0.1674 | 0.6348 |
| ptp-N4 | 9 | 4 | 56 | 61 | 0.078 | 0.2536 | 0.3076 |
| **Total** | **211** | **163** | **2100** | **1673** |  |  |  |
