# Supplementary material for: Analysis of Genetic Variation across the Encapsidated Genome of Microplitis demolitor Bracovirus in Parasitoid Wasps
Source: PLoS One. 2016 Jul 8;11(7):e0158846. doi: 10.1371/journal.pone.0158846 (PMC4938607; doi:10.1371/journal.pone.0158846)
Supplement: S6 Table — (DOCX) [file pone.0158846.s008.docx]

Supplementary Table 1. Library construction and sequencing parameters used to sequence each sample of BV proviral DNA.

| **Sample name** | **Number of individuals sampled** | **Average library size** | **Library construction method** | **Number of sequencing cycles** | **Illumina sequencing system** | **Sequencing location** | **Accession numbers** |
| --- | --- | --- | --- | --- | --- | --- | --- |
| Pooled field | 12 | 298 | TruSeq DNA | 150 | miSeq | UGA GGF* | SRR3420509 |
| Individual field | 1 | 150 | Nextera XT DNA | 150 | miSeq | UGA GGF | SRR3420511 |
| Pooled laboratory 1 | 15 | 342 | TruSeq DNA | 100 | HiSeq | Hudson Alpha | SRR3420508 |
| Pooled laboratory 2 | 30 | 363 | TruSeq DNA | 100 | miSeq | UGA GGF | SRR3420507 |
| MmBV | 100 | 400 | TruSeq DNA | 100 | HiSeq | Hudson Alpha | KX223693-KX223748 |

*The Georgia Genomics Facility at the University of Georgia
